# Linking NrfD/PsrC‐like architecture to energy conservation: Functional residues in the quinone reactive QrcABCD complex of sulfate‐reducing bacteria

**DOI:** 10.1002/pro.70557

**Published:** 2026-04-08

**Authors:** Gonçalo Manteigas, Teresa Catarino, João B. Vicente, Américo G. Duarte, Inês A. C. Pereira

**Affiliations:** ^1^ Instituto de Tecnologia Química e Biológica António Xavier Universidade Nova de Lisboa Oeiras Portugal; ^2^ Departamento de Química, Faculdade de Ciências e Tecnologia Universidade Nova de Lisboa Caparica Portugal

**Keywords:** anaerobic respiration, energy conservation, membrane complex, NrfD/PsrC family, redox loop, sulfate reduction

## Abstract

The QrcABCD quinone reductase complex is an electrogenic complex present in sulfate‐reducing bacteria of the *Desulfobacterota* phylum. It operates as a cytochrome *c*_3_:menaquinone oxidoreductase involved in electron transfer from periplasmic hydrogen or formate oxidation to the menaquinone (MK) pool. Two proteins in this complex, QrcC and QrcD, form a redox dimer (QrcCD) responsible for MK reduction coupled to proton uptake from the cytoplasm. QrcD belongs to the NrfD/PsrC family, and homologs are found in many bacterial redox complexes in different bioenergetic contexts. In this work a homologous overexpression system for QrcABCD was established in *Nitratidesulfovibrio vulgaris* Hildenborough and used to produce variants with changes in key amino acids proposed to be involved in energy conservation. Growth studies of the modified strains combined with activity assays with isolated protein variants reconstituted in proteoliposomes revealed the essential role of key amino acids involved in the MK‐binding site on the P‐side of the membrane, and as part of a proposed proton uptake channel from the cytoplasm to the MK‐binding site. The results support the proposed model for energy conservation where, upon formate or hydrogen oxidation, QrcABCD is involved in a redox‐loop mechanism with another membrane complex, generating *pmf* by proton and electron uptake from different sides of the membrane, without active proton pumping.

## 1 INTRODUCTION

Respiratory membrane complexes are essential to generate and maintain a protonmotive force (*pmf*) across the membrane by performing redox reactions that are coupled to charge transfer. This can involve direct proton pumping, a Q‐cycle, or a redox loop mechanism involving membrane proteins facing opposite sides of the membrane (Mitchell 1961; Sarewicz et al. 2021; Simon et al. 2008; Wikström et al. 2015). In prokaryotes, anaerobic respiration is associated with a wide range of diverse membrane redox complexes involved in energy conservation, in contrast to the well‐known complexes involved in mitochondrial and prokaryotic aerobic respiration. The complexes of anaerobic respiration are characterized by a highly modular character, where a limited set of early‐evolving protein redox modules are found in different organizations, giving rise to different biological functions (Baymann et al. 2003; Grein et al. 2013). This feature is particularly striking in the dissimilatory metabolism of sulfur compounds (Grein et al. 2013), which was among the early biological strategies to obtain energy. In the widespread process of sulfate respiration, three main membrane complexes are involved in energy transduction: the QrcABCD (Duarte et al. 2018; Venceslau et al. 2010), the QmoABC (Pires et al. 2003; Ramos et al. 2012), and the DsrMKJOP complexes (Barbosa et al. 2024; Pires et al. 2006), all of which include different combinations of such redox modules. Sulfate‐reducing prokaryotes are key organisms in anoxic environments, which set in motion the biogeochemical sulfur cycle and are also key drivers of the carbon cycle, namely in marine sediments (Diao et al. 2023; Rabus et al. 2015).

The quinone reductase complex (QrcABCD) is highly conserved in cytochrome‐rich sulfate‐reducing bacteria of the *Desulfobacterota* phylum (Pereira et al. 2011; Rabus et al. 2015; Venceslau et al. 2010), where it links periplasmic formate and H_2_ oxidation to reduction of the menaquinone pool in the membrane (Venceslau et al. 2010), and it was the first membrane complex shown to conserve energy in these bacteria (Duarte et al. 2018). QrcABCD is essential for growth with either formate or H_2_ as energy sources (Keller et al. 2014; Li et al. 2009), and is also important for their production under syntrophic growth conditions (Meyer et al. 2013; Walker et al. 2009). The QrcABCD complex is composed of four subunits, the multiheme cytochrome *c* QrcA, and the QrcBCD subunits that are related to the complex iron–sulfur molybdoenzyme (CISM) family (Grimaldi et al. 2013). The largest subunit, QrcB, is related to the molybdopterin catalytic subunits of CISM enzymes but does not have any redox cofactors and therefore no enzymatic activity. QrcC binds four FeS clusters and QrcD is an integral membrane protein with 10 transmembrane helices and no cofactors, which interacts with quinones (Grimaldi et al. 2013). QrcABCD is the physiological electron acceptor of the abundant periplasmic Type I cytochrome *c*_*3*_ (TpI*c*_*3*_) and it was shown that it can form a supercomplex with TpI*c*_*3*_ and the membrane‐bound [NiFeSe] hydrogenase (Venceslau et al. 2011), working in a redox loop mechanism together with other respiratory complexes essential for sulfate respiration, namely QmoABC and DsrMKJOP (Duarte et al. 2018). Electron transfer (ET) experiments performed with reduced TpI*c*_3_ and QrcABCD reconstituted in proteoliposomes containing menaquinone, demonstrated that QrcABCD is electrogenic, consuming electrons and protons from opposite sides of the membrane, with a H^+^/e^−^ ratio of 1 (Duarte et al. 2018). The key proteins in this mechanism are QrcC and QrcD, which together compose a redox module that is responsible for the quinone redox chemistry. The QrcCD dimer is a member of the NrfCD proteins (Calisto and Pereira 2021; Duarte et al. 2021; Simon and Kern 2008), with homologs widespread in diverse complexes across Bacteria and Archaea, involved in different types of metabolism converting hydrogen, oxygen, nitrogen and sulfur compounds, arsenate or organohalides, where they are associated with electroneutral, electrogenic or energy‐driven reactions (Duarte et al. 2018; Duarte et al. 2021). In this family, the integral membrane subunit (NrfD/PsrC‐like) has 8–10 transmembrane helices (TMH) and is associated with a soluble electron transfer subunit with up to four iron–sulfur (FeS) clusters (NrfC/PsrB) (Duarte et al. 2021). A quinone binding site (QBS) in the NrfD/PsrC membrane subunit has been characterized in the structures of the polysulfide reductase (PsrABC) (Jormakka et al. 2008) and the alternative complex III (ACIII) (Xin et al. 2024) and proposed for several others (Dietrich and Klimmek 2002; Duarte et al. 2018; Duarte et al. 2021; Geijer and Weiner 2004; Lubek et al. 2019; Shi et al. 2020; Sun et al. 2018; Wu et al. 2025). In the majority of cases, this QBS faces the positive side (P‐side) of the membrane, at the interface between this subunit and the NrfC/PsrB FeS cluster subunit. The NrfD/PsrC protein has been proposed to perform proton pumping (Beaton et al. 2018; Pinske et al. 2015; Sousa et al. 2018; Sun et al. 2018), be energy‐driven (Dietrich and Klimmek 2002; Duarte et al. 2021; Simon et al. 2008) or eletroneutral (Bogachev et al. 1996; Duarte et al. 2021; Simon et al. 2008), but experimental evidence for electrogenicity has so far been obtained only for the QrcABCD complex, which was shown to generate a *pmf* without proton pumping, by obtaining protons and electrons for menaquinone reduction from opposite sides of the membrane (Duarte et al. 2018).

In previous work, a homology‐based model was generated for QrcABCD, which allowed us to propose conserved amino acids that could be involved in quinone interaction and in proton transfer from the putative QBS to the cytoplasm (Duarte et al. 2018). In this work, a deletion strain lacking the *qrcABCD* genes was constructed in *Nitratidesulfovibrio vulgaris* Hildenborough (formerly *Desulfovibrio vulgaris* Hildenborough) and complemented with the complete *qrc* operon to generate a homologous expression system to produce recombinant QrcABCD (rQrcABCD). This system was used to produce single amino acid variants of rQrcABCD targeting specific residues among those predicted to be involved in quinone binding and proton transfer. Growth studies combined with kinetic assays with QrcABCD variants reconstituted in menaquinone‐4 (MK4) containing proteoliposomes revealed several essential residues for the QrcABCD molecular mechanism.

## 2 RESULTS

### 2.1 Expression system for production of rQrcABCD and variants

A homologous expression system to produce rQrcABCD was developed by first deleting the *qrcABCD* operon in *N. vulgaris* Hildenborough through the insertion of a kanamycin resistance cassette by homologous recombination with a suicide plasmid. This chromosomal region was amplified by PCR in both the wild‐type strain (WT) and knock‐out strains to confirm *qrc* operon deletion (Figure S1, Supporting information). The *ΔqrcABCD* strain was then complemented with the complete *qrcABCD* operon cloned in an expression vector, under the control of a constitutive promoter and carrying a Strep‐tag at the 3′ side of *qrcD* (pMOGM2‐*qrcABCD*‐strep‐tag), generating the *ΔqrcABCD*:pMOGM2‐*qrcABCD*‐strep‐tag strain expressing the rQrcABCD complex (named rQrc strain). To confirm the effect of deleting the *qrcABCD* genes in *N. vulgaris* Hildenborough, growth of the *ΔqrcABCD* and rQrc strains was compared with the WT in growth with lactate, pyruvate, H_2_ or formate and sulfate as electron acceptor (Figure S2). The *ΔqrcABCD* strain was capable of growing in lactate/sulfate with a doubling time and growth rates identical to the WT (Table S1 and Figure S2a), whereas it could not grow with formate, H_2_ or pyruvate and sulfate (Table S1 and Figure S2b–d). This confirmed that the QrcABCD complex is essential for *N. vulgaris* growth with the periplasmic electron donors H_2_ and formate, as previously reported for the sulfate reducer *Oleidesulfovibrio alaskensis* G20 (previously named *Desulfovibrio desulfuricans* G20) (Keller et al. 2014; Li et al. 2009). Complementation of the knock‐out strain with the *qrcABCD* genes in trans (strain rQrc) restored the phenotype ability to grow under formate/sulfate, H_2_/sulfate or pyruvate/sulfate (Figure S2).

**FIGURE 1:**
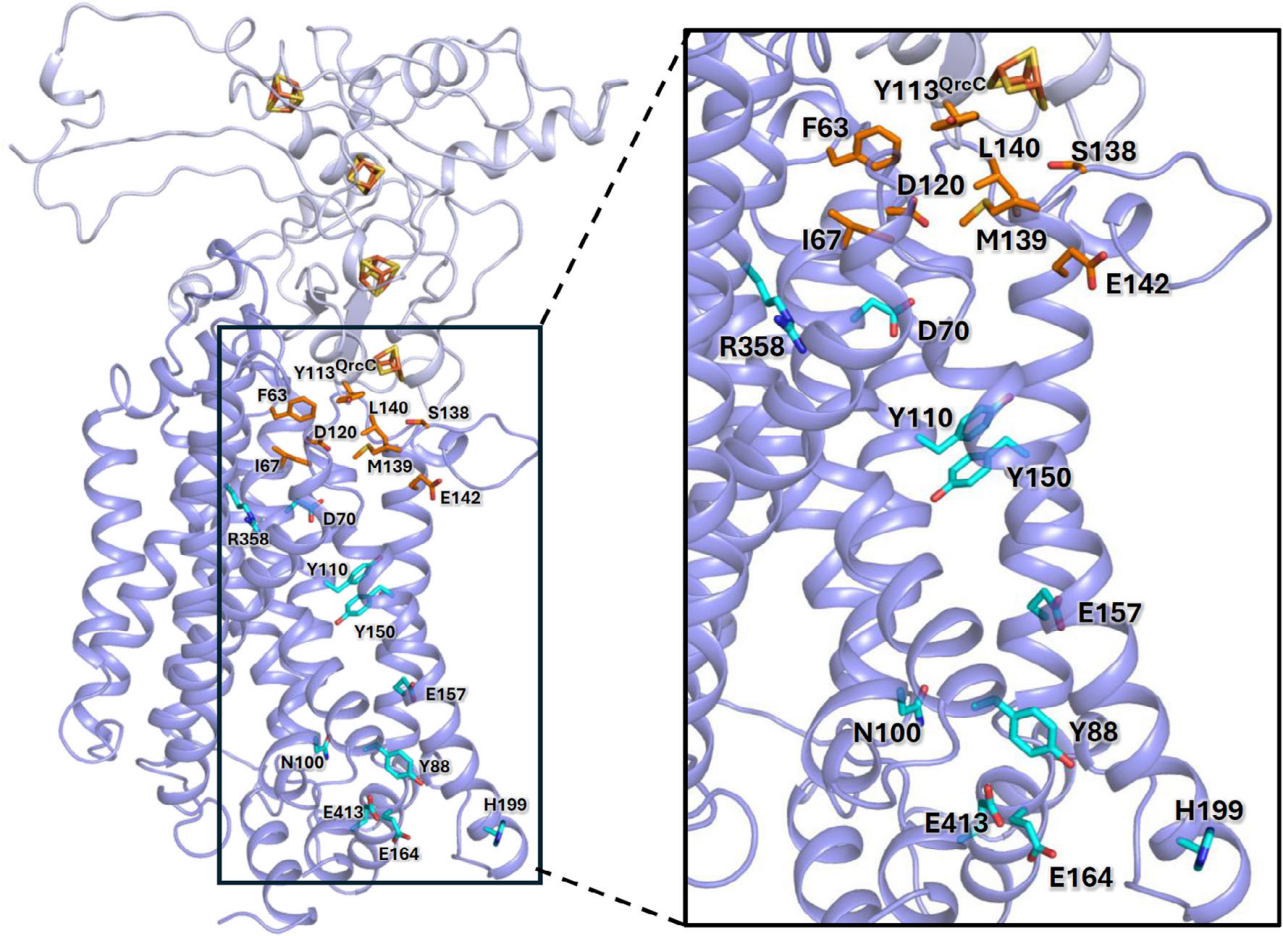
Structural model of QrcCD. Overall view of the QrcCD model obtained using MODELLER (Duarte et al. 2018), showing the four FeS clusters in QrcC and conserved residues proposed to be involved in the QBS (in orange) and proton transfer (in cyan). Figure prepared with PyMol.

A previous structural model of the QrcCD subunits highlighted several conserved residues that could be involved in quinone binding and in a proton transfer pathway (Figure 1) (Duarte et al. 2018). Of these, we selected eight residues that were modified through site directed mutagenesis to evaluate their role in catalysis by rQrcABCD. The modifications targeted residues in the putative QBS, namely Y113^QrcC^, D120^QrcD^, and S138^QrcD^, and along the putative QrcD H^+^ pathway, D70^QrcD^, Y110^QrcD^, Y150^QrcD^, R358^QrcD^, and E413^QrcD^ (Figure 1). All these residues were replaced with Ala, whereas for two residues two other mutations were also analyzed, namely D70H^QrcD^ and Y113F^QrcC^. The plasmids carrying these mutations were transformed into the *ΔqrcABCD* strain to generate variant strains that were tested for in vivo growth profiling (for simplicity, only formate was tested as periplasmic electron donor) and used for the isolation of the respective QrcABCD variants for in vitro experiments with the reconstituted proteins in liposomes, to assess the physiological catalytic activity with TpI*c*_3_ and MK4.

### 2.2 Physiological impact of the QrcCD mutations

**FIGURE 2:**
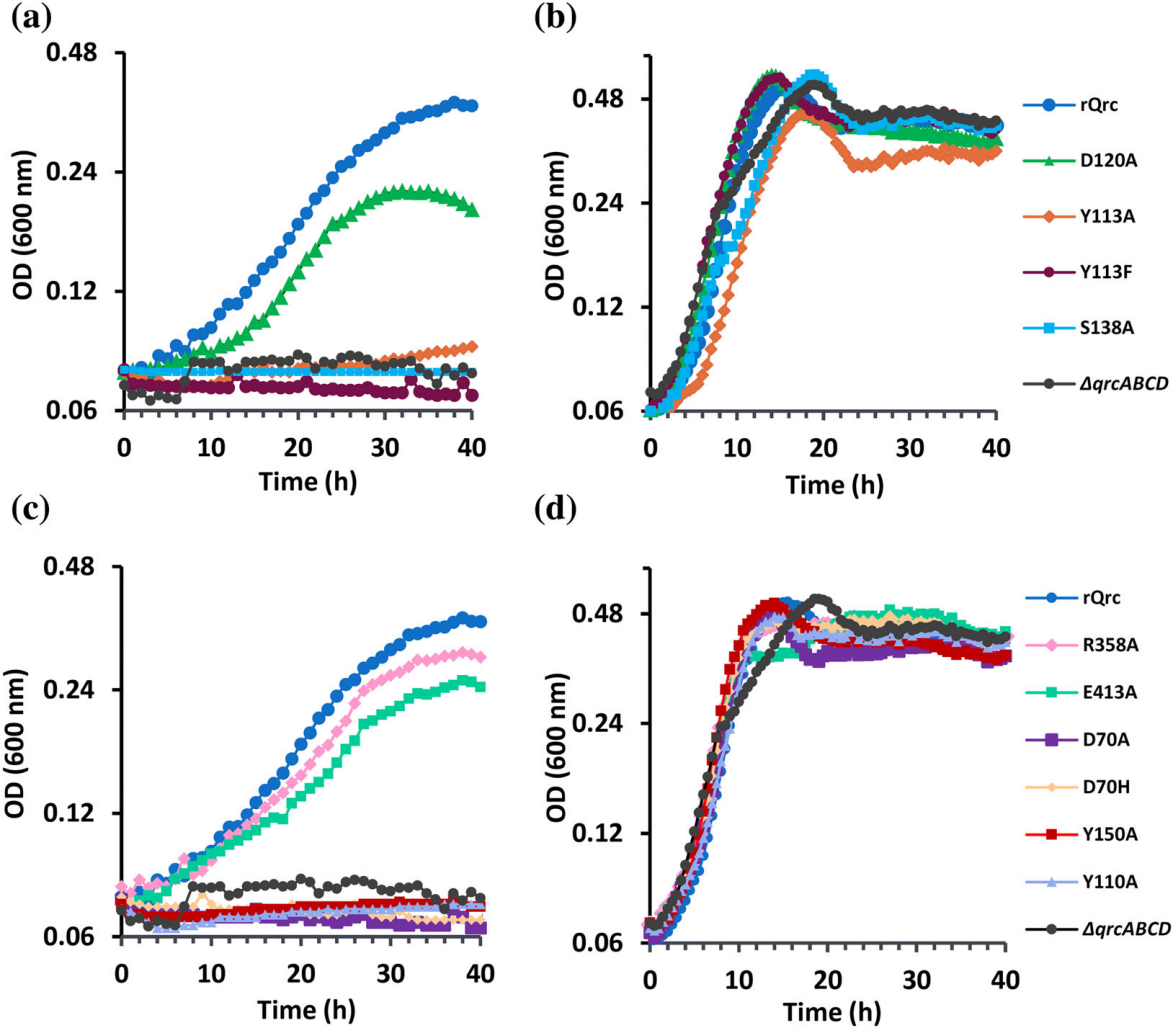
Growth profiles of *N. vulgaris* strains expressing rQrcABCD variants. Growth curves of rQrc strain, *ΔqrcABCD*, and variants in residues from the QBS (a, b) and the proposed proton pathway (c, d). Growth on formate/sulfate (a, c) and lactate/sulfate (b, d). Each point is the average of three independent biological replicates.

The 10 constructed strains, carrying the Y113A^QrcC^, Y113F^QrcC^, D70A^QrcD^, D70H^QrcD^, D120A^QrcD^, S138A^QrcD^, Y150A^QrcD^, Y110A^QrcD^, R358A^QrcD^, and E413A^QrcD^ mutations, were grown in defined media with lactate or formate as electron donors and sulfate as electron acceptor, to evaluate the effect of the mutations in vivo (Figure 2). The growth curves reveal that the mutations Y113A^QrcC^, Y113F^QrcC^, S138A^QrcD^, D70A^QrcD^, D70H^QrcD^, Y150A^QrcD^, and Y110A^QrcD^ prevent bacterial growth on formate/sulfate, pointing to a defect in QrcABCD function, while modifications E413A^QrcD^, D120A^QrcD^, and R358A^QrcD^ had a marginal effect on growth rates and duplication times, but with D120A^QrcD^ reaching a lower maximal cell density (Figure 2a and Table S2). Like the rQrc strain, all the modified strains could grow on lactate/sulfate with similar growth rates and duplication times (Figure 2b,c and Table S2).

### 2.3 Production of the rQrcABCD variants

The engineered strains were used to produce rQrcABCD complex and variants. The recombinant proteins were solubilized from the membranes using the detergent n‐dodecyl‐β‐D‐maltoside (DDM) and purified by Strep‐tag affinity chromatography. The purified rQrcABCD had an identical electrophoretic profile as the native QrcABCD (Venceslau et al. 2010), with all four subunits visible in a denaturing PAGE (Figure S3a). The presence of the Strep‐tag at the C‐terminus of rQrcD was confirmed by Western blot both in denaturing and native PAGE, where a single band corresponding to the whole rQrcABCD complex (approx. 230 kDa) was observed (Figure S3b). The different variants were expressed and purified using the same procedure, presenting similar electrophoretic profiles (Figure S4). The rQrcABCD (and variants) also showed UV–vis spectra identical to the native complex (Figure S5). This expression system allowed fast purification of rQrcABCD and variants with a yield ranging from 0.15 to 0.3 mg per g cells (wet weight), depending on the variant. To evaluate the stability of the different variants, their melting temperature was determined using differential scanning fluorimetry (DSF). QrcABCD contains a total of 38 Trp residues, yet no consistent intrinsic tryptophan fluorescence‐based DSF data were obtained, probably due to fluorescence quenching by the hemes and by detergent since several Trp are located in transmembrane helices of QrcD. DLS data were also likely affected by the strong heme absorption at the fixed laser wavelength (412 nm). Therefore, thermal stability was evaluated by measuring the turbidity profile with increasing temperature, which exhibited the expected sigmoidal curve, with *T*_turb(50)_ representing the temperature at which turbidity reaches its half‐maximal value. Under these conditions, rQrcABCD had a *T*_turb(50)_ of 57.2 ± 0.9°C, and the variants presented similar or only slightly decreased stability (<5°C) (Figure S6). The D70A^QrcD^ and R358A^QrcD^ were the most affected variants, with ~3–4°C lower *T*_turb(50)_, while the other mutations had a null to mild impact on stability (~2°C lower *T*_turb(50)_).

### 2.4 The role of conserved residues in QrcCD redox mechanism

**FIGURE 3:**
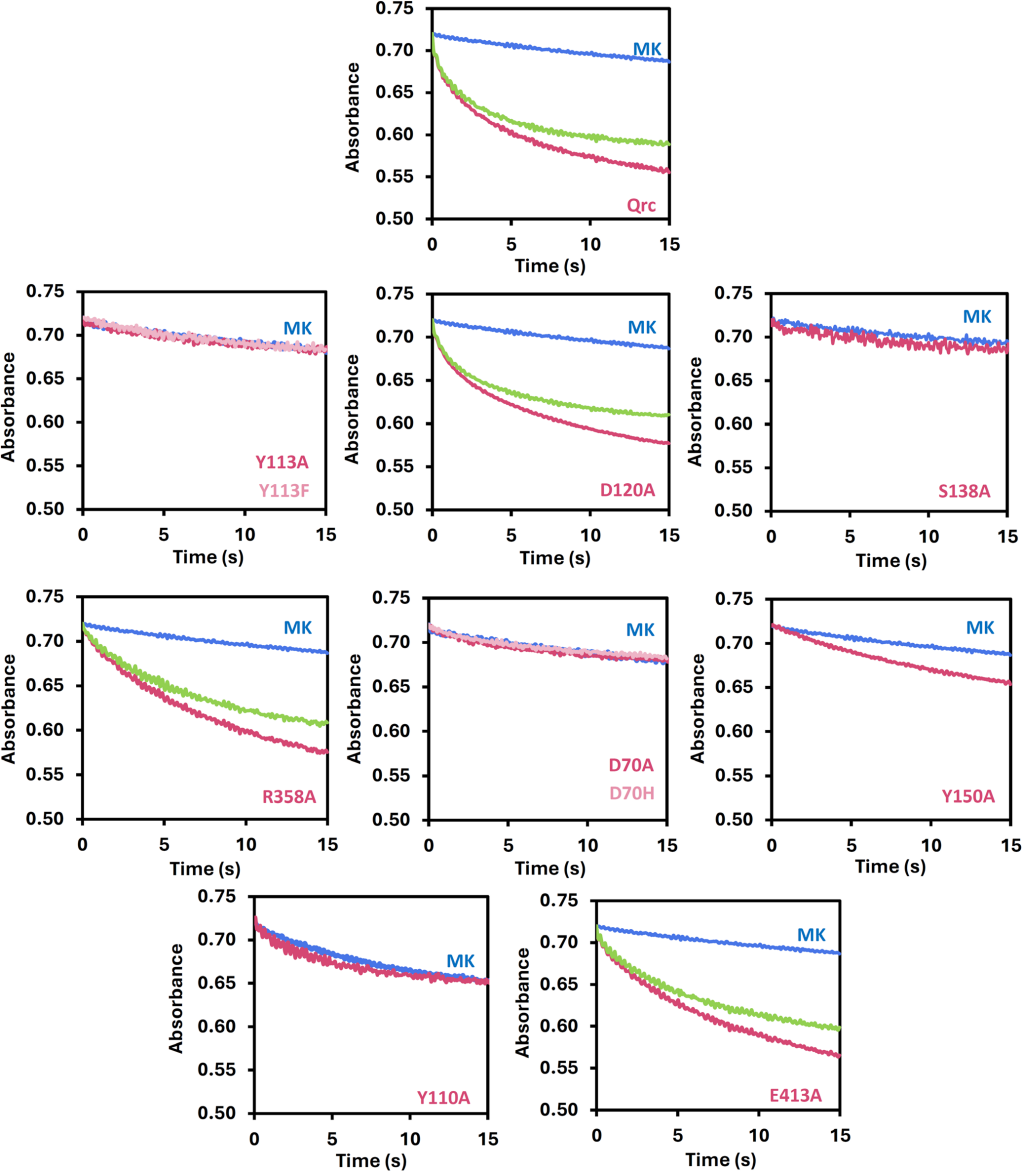
Electron transfer experiments with rQrcABCD and variants reconstituted in MK4‐containing liposomes. Traces show absorbance changes at 552 nm corresponding to the oxidation of pre‐reduced TpI*c*_3_ after mixing with distinct proteoliposome suspensions. Unspecific oxidation of TpI*c*_3_ by MK4 is shown in blue (MK), total oxidation of TpI*c*_3_ by rQrcABCD variants and MK4 are shown in magenta and pink, and the subtraction between the two, corresponding to the rate of TpI*c*_3_ oxidation due to rQrcABCD activity is shown in green.

In a previous study, the native QrcABCD complex was shown to be electrogenic, with a proton‐to‐electron ratio of 1, in experiments where the protein was reconstituted in menaquinone containing liposomes (Duarte et al. 2018). To assess the impact of the selected mutations, the produced variants were similarly reconstituted in MK4‐containing liposomes and further used in ET experiments with the physiological partner, TpI*c*_3_. In selected experiments, phenol red was placed inside the liposomes as a pH probe. The TpI*c*_3_ cytochrome was pre‐reduced with dithionite to about 95% (to prevent excess of reductant) and reacted with the different proteoliposomes using stopped‐flow equipment inside an anaerobic chamber. ET was monitored through the re‐oxidation rate of TpI*c*_3_, following changes in absorbance at 552 nm, while the protonation state of the phenol red inside the vesicles was monitored at 560 nm (a TpI*c*_3_ isosbestic point). In these experiments, a glucose oxidase/catalase O_2_ scavenging system was added to the working solution to eliminate any traces of residual oxygen, preventing any possible TpI*c*₃ reoxidation by low levels of O_2_. The rQrcABCD showed similar behavior to the previous study (Duarte et al. 2018), catalyzing the fast oxidation of TpI*c*_3_ with MK4 (Figure 3), while MK4‐containing liposomes without QrcABCD showed the unspecific ET between the cytochrome and the MK4 in the liposomes, as also previously reported (Duarte et al. 2018). This unspecific ET needs to be subtracted to determine the specific ET from TpI*c*_3_ to QrcABCD. The variants Y113A^QrcC^, Y113F^QrcC^, and S138A^QrcD^ of the putative QBS, and D70A^QrcD^, D70H^QrcD^, Y110A^QrcD^, Y150A^QrcD^ of the putative H^+^ pathway, displayed no significant ET to MK4 (Figure 3). In contrast, the variants D120A^QrcD^ (QBS), R358A^QrcD^, and E413A^QrcD^ (H^+^ pathway) presented a specific ET between TpI*c*_3_ and QrcABCD that is significantly higher than the unspecific oxidation rate of TpI*c*_3_ directly by MK4 (Figure 3). For these variants, the changes in phenol red absorption were also measured in the same time frame, to monitor the internal pH of the liposomes (Figure S7). The absorption changes were calibrated using the irreversible tryptic hydrolysis of N‐α‐tosyl‐L‐arginyl‐O‐methylester (TAME) (Figure S8).

**FIGURE 4:**
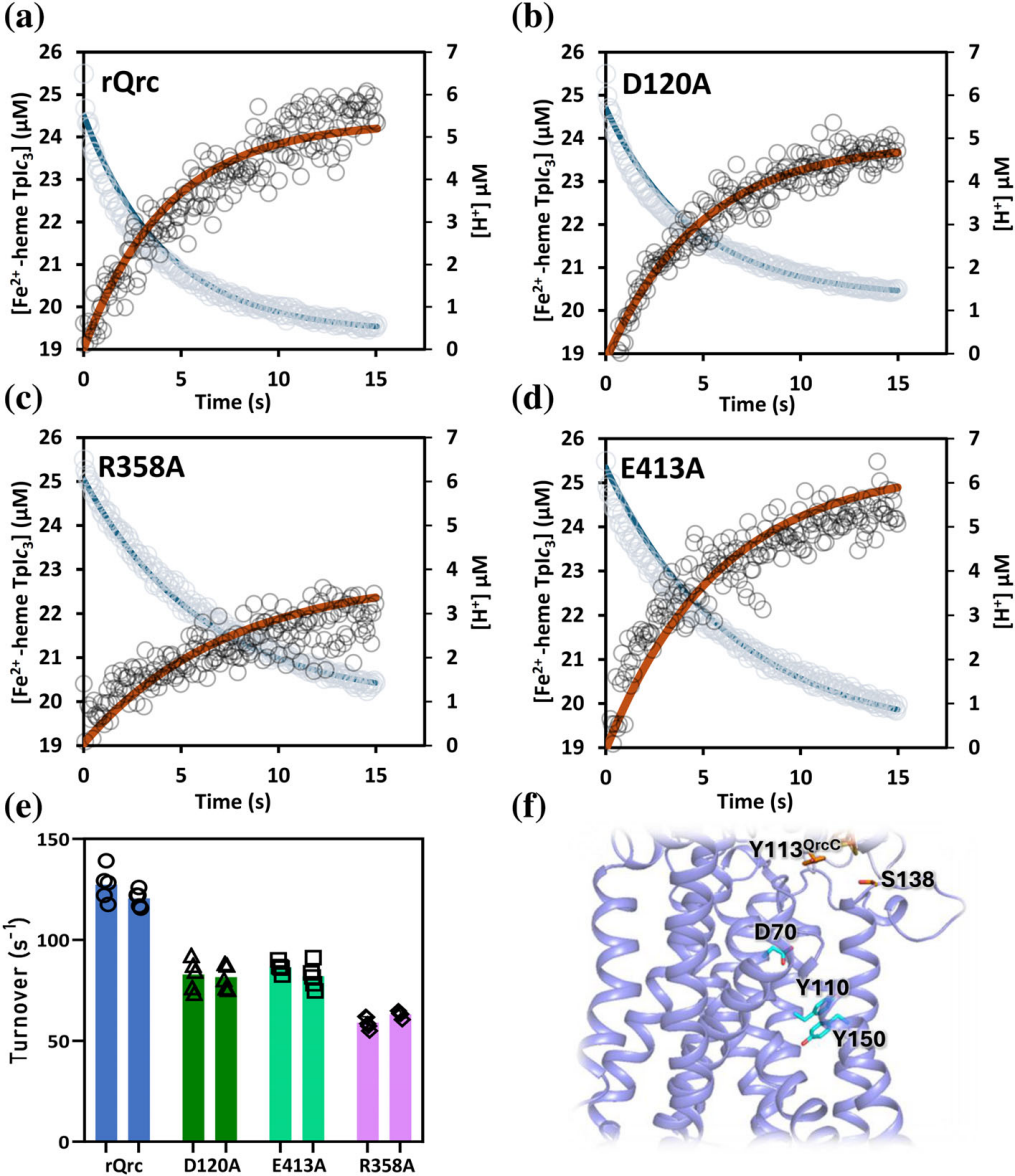
Electron and proton transfer observed in proteoliposomes with rQrcABCD and variants. (a) rQrcABCD; (b) D120A^QrcD^; (c) R358A^QrcD^; (d) E413A^QrcD^. Reduced heme concentration (blue circles) and proton concentration (black circles) after mixture of reduced TpI*c*_3_ and each of the proteoliposome suspensions. Data points were fitted with single exponentials: heme oxidation (blue lines), and proton uptake (brown lines). (e) TpI*c*_3_ oxidation rates of rQrcABCD and variants in proteoliposomes (data from two biological (columns) and five technical replicates (dots)). rQrcABCD (blue), D120A^QrcD^ (green), E413A^QrcD^ (light green), R358A^QrcD^ (pink); (f) Close‐up view of QrcCD model highlighting the essential residues for the energy conservation mechanism.

The changes in reduced TpI*c*_3_ and phenol red absorbance were converted to concentration of reduced heme (corresponding to one electron) and concentration of H^+^ for rQrcABCD and D120A^QrcD^, R358A^QrcD^, and E413A^QrcD^ variants (Figure 4). These results show that for the rQrcABCD proteoliposomes electrons are consumed with a rate constant (*k* ^e−^) of 0.24 s^−1^ with a maximum concentration of oxidized heme of 5.1 μM, while protons are taken up from the vesicles' inner compartment in the same time scale, following an inverse single exponential profile that can be fitted with similar kinetic parameters: *k* ^H+^ = 0.23 s^−1^ and A^H+^ = 5.3 μM, confirming the H^+^/e^−^ of 1, determined either from the rate constants or the concentration of electrons and protons used for MK4 reduction (Table 1). The data also allow us to calculate the rQrcABCD turnover to be 120 ± 8 s^−1^, which is similar to the previously reported value (147 s^−1^) (Duarte et al. 2018). The same parameters were also calculated for the variants D120A^QrcD^ (QBS), R358A^QrcD^ and E413A^QrcD^ (H^+^ pathway) showing somewhat lower turnover and electron and proton transfer rates (Table 1). When compared to rQrcABCD, these variants display 68, 53 and 68% of the enzymatic activity, respectively (Figure 4e).

**TABLE 1:** Kinetic parameters of electron transfer and proton uptake by rQrcABCD and variants reconstituted in proteoliposomes.

	*k*_e−_ (s^−1^)	[e^−^] (μM)	*k*_H_+ (s^−1^)	[H^+^] (μM)	Turnover (s^−1^)	*k*_H+_/*k*_e−_	[H^+^]/[e^−^]
rQrcABCD	0.24	5.1	0.23	5.3	120 ± 8	1.0	1.0
D120A^QrcD^	0.22	4.4	0.22	4.7	82 ± 6	1.0	1.1
R358A^QrcD^	0.16	5.1	0.13	3.8	63 ± 2	0.8	0.8
E413A^QrcD^	0.15	6.1	0.17	6.4	82 ± 6	1.1	1.0

*Note*: Each kinetic trace was fitted with a single exponential *A* × exp^(−*k*×*t*)^, where *k* corresponds to the rate constant and *A* to the exponential amplitude, which gives the concentration of electrons or protons exchanged in the process.

## 3 DISCUSSION

The widespread sulfate‐reducing bacteria from the *Desulfobacterota* phylum are characterized by the presence of uptake periplasmic hydrogenases and formate dehydrogenases that are soluble and lack a membrane subunit for direct quinone reduction (Pereira et al. 2011). Instead, these enzymes use as electron acceptor the abundant tetraheme cytochrome *c*, TpI*c*_3_, which then feeds electrons to the QrcABCD membrane complex that catalyzes menaquinone reduction (Duarte et al. 2018; Venceslau et al. 2010). The QrcABCD is essential for growth on periplasmic electron donors such as H_2_ or formate, but not on cytoplasmic ones like lactate (Keller et al. 2014; Li et al. 2009), and is electrogenic by drawing electrons and protons from opposite sides of the membrane (Duarte et al. 2018). In this work, a QrcABCD expression system was developed, allowing its production and fast purification along with the production of variants. Ten single amino acid modifications were made, targeting eight conserved residues in the QrcCD redox module that were predicted to have a key role in the energy conserving mechanism (Duarte et al. 2018). Modifications in Y113^QrcC^ and S138^QrcD^ of the QBS, and D70^QrcD^, Y110^QrcD^, and Y150^QrcD^ of the proposed H^+^ pathway, fully compromise the physiological function of QrcABCD, as the genetically modified *N. vulgaris* strains carrying single mutations of these residues could not couple formate oxidation to sulfate respiration, but grew normally on lactate/sulfate. Further biochemical experiments with the corresponding purified variants reconstituted in MK4‐containing liposomes also showed no ET or H^+^ uptake. On the other hand, strains carrying the mutations D120A^QrcD^, R358A^QrcD^, and E413A^QrcD^ could still grow with formate/sulfate, and the corresponding variants showed in vitro catalytic activity.

Comparable site‐directed mutagenesis studies have been reported for three homologs from the NrfD/PsrC family: *Escherichia coli* type‐2 hydrogenase (Hyd‐2/HybOABC) (Lubek et al. 2019), DMSO reductase (DmsABC) (Rothery and Weiner 1996), and *Wolinella succinogenes* polysulfide reductase (PsrABC) (Dietrich and Klimmek 2002). In addition, structural information for members of this family is available for the phylogenetically distant archaeon *Thermus thermophilus* PsrABC (Jormakka et al. 2008), and for several bacterial Alternative Complex III (Shi et al. 2020; Sousa et al. 2018; Sun et al. 2018; Wu et al. 2025; Xin et al. 2024). Regarding the QBS, we tested modifications in Y113^QrcC^, D120^QrcD^, and S138^QrcD^. Y113^QrcC^ is the only amino acid from QrcC predicted to be involved in the QBS. Its substitution with an Ala or a Phe totally inhibits electron transfer to the quinone pool, suggesting that the Y113 hydroxyl group plays a crucial role in the reaction with MK4. Y113^QrcC^ is present between two Cys ligands of the proximal FeS cluster and the structural model predicts its side chain to be on top of the QBS, above the quinone, shielding it from direct contact with the periplasm (Figure 1). Y113^QrcC^ is broadly conserved in the NrfC/PsrB family with some exceptions, namely in *T. thermophilus* PsrABC where an Ala is present instead (Jormakka et al. 2008), but its role was not previously tested in any protein. S138^QrcD^ is highly conserved in the NrfD/PsrC family (Calisto and Pereira 2021), and in the QrcCD model is positioned close to Y113^QrcC^ in the QBS. The substitution of S138^QrcD^ for an Ala prevents ET from TpI*c*_3_ to the MK4 pool. Modification of the equivalent residue in *W. succinogenes* PsrC (S94) (see structural prediction by AlphaFold; Figure S9) also decreased its enzymatic activity (Dietrich and Klimmek 2002), whereas in *E. coli* Hyd‐2, altering the corresponding HybB S129 did not significantly affect H_2_ oxidation activity (Lubek et al. 2019). The third residue of the QBS to be mutated, D120^QrcD^, is predicted in the model to be close to the QBS (Figure 1). Its exchange for an Ala allows for growth on formate, albeit to a lower cell density, and to about 66% in vitro activity relative to the WT, revealing it does not play a critical role in the QBS. Curiously, modifications in the equivalent residues in *W. succinogenes* PsrC D76N/H/L (Dietrich and Klimmek 2002) or in the *E. coli* DmsC H65 to an Arg (Geijer and Weiner 2004) fully inactivated the enzymes, suggesting that the QBS in QrcD may be slightly different since it is able to accommodate this change.

In terms of the proposed H^+^ pathway from the cytoplasm to the quinone binding site, residues D70^QrcD^, Y110^QrcD^, Y150^QrcD^, R358^QrcD^, and E413^QrcD^ were mutated. D70^QrcD^ was suggested to connect the putative QBS to the proton pathway (Duarte et al. 2018), and was here confirmed to be a key residue for the function of QrcABCD as its replacement for an Ala or a His did not allow for growth on formate or in vitro ET activity, indicating that the negatively charged carboxylate in this position is essential for the mechanism. Substitution of the equivalent Y23 to a Phe in *W. succinogenes* PsrC also caused full inhibition of polysulfide respiration (Dietrich and Klimmek 2002), while the equivalent mutation D58A in *E. coli* HybB resulted in loss of MK reduction under H_2_ oxidation, while H_2_ evolution from glycerol was still possible (Lubek et al. 2019). The substitutions of Y110^QrcD^ or Y150^QrcD^ for Ala also led to full inactivation of QrcABCD, both in terms of growth on formate and in vitro activity, suggesting an essential role in H^+^ transport from the cytoplasm to the QBS. In the structural model, Tyr 110 and 150 are in the middle of the predicted H^+^ pathway, 10‐Å apart, and water molecules may be involved in H^+^ transport between them. The residue Y99 of *E. coli* HybB (equivalent to Y110^QrcD^) was also shown to be essential for H^+^ uptake from the cytoplasm in H_2_ oxidation (Lubek et al. 2019), whereas for Y106 of *W. succinogenes* PsrC (equivalent to Y150^QrcD^), mutating this residue did not have a strong impact on growth or activity (Dietrich and Klimmek 2002). Polysulfide reduction by menaquinol is an energetically unfavorable reaction, most likely driven by the *pmf*, suggesting that quinol oxidation involves proton release to the cytoplasm. In turn, hydrogen oxidation by Hyd‐2 coupled to menaquinone reduction is highly favorable, and HybB is phylogenetically closer to QrcD, and so it may function in a similar way, as supported by the observed loss of activity from mutations in its putative channel (Lubek et al. 2019), although a proton‐pumping mechanism cannot be excluded. The mutation of E413^QrcD^ had a low impact on growth with formate, with slightly lower doubling time and final cell density, while the in vitro activity of the respective QrcABCD protein was about 68% of the WT. This indicates a possible role for this residue, although not essential. In the structural model, E413^QrcD^ is predicted to be close to the cytoplasm and most likely exposed to the solvent (Figure 1), indicating that a water molecule or another protonable residue in the vicinity can easily take its place. The QrcABCD complex has a similar, albeit simpler, composition to the ACIII complex, for which several structures are available (Shi et al. 2020; Sousa et al. 2018; Sun et al. 2018; Wu et al. 2025; Xin et al. 2024). The ACIII complex catalyzes the reverse reaction to QrcABCD, the reduction of a periplasmic cytochrome *c* with electrons from the quinol pool, and is proposed to perform proton pumping (Duarte et al. 2018; Shi et al. 2020; Sousa et al. 2018; Xin et al. 2024), although this has not yet been experimentally confirmed. For QrcABCD, no proton pumping across the membrane could be detected, with only proton uptake from the cytoplasm for menaquinone reduction at the P‐side being observed (Duarte et al. 2018). Several residues in the ACIII membrane subunit ActC were proposed to operate in proton translocation. These are not strictly conserved for other members of the NrfD family where alternative residues could perform a similar function (Calisto and Pereira 2021; Shi et al. 2020; Xin et al. 2024). Among these residues, an Arg (R395^ActC^ in *Rhodothermus marinus* ACIII) was proposed to play a key role as a gatekeeper in proton translocation (Calisto and Pereira 2021; Shi et al. 2020), and to be conserved for all members of the NrfD family (Calisto and Pereira 2021). An important role for an Arg is also present in F‐type ATP synthases where a strictly conserved Arg in the long horizontal a‐subunit helix acts as an electrostatic barrier, preventing backward rotation of F_0_ domain and proton leakage (Kühlbrandt and Davies 2016). Mutation of this a‐subunit Arg to a smaller or negatively charged residue resulted in unproductive dissipation of the proton gradient (Mitome et al. 2010). When the equivalent residue to R395^ActC^ in QrcD, R358^QrcD^, was replaced with Ala this did not have a significant impact on growth by formate‐sulfate respiration, indicating that this residue does not have an essential role under these conditions. In the proteoliposome experiments the R358A^QrcD^ variant had about 50% of the WT activity and lower ET and H^+^ transport rates (Figure 4 and Table 1). The H^+^/e^−^ ratio is 0.8, suggesting a small defect in H^+^ uptake from the cytoplasm towards the QBS, which may originate from some structural instability, evidenced by the lower thermostability (Figure S6). Overall, the results do not suggest a key role for this residue in gating H^+^ pumping by two half channels in QrcD, as previously proposed for ACIII ActC and other members of the NrfD family (Calisto and Pereira 2021).

In conclusion, this work supports the proposed essential role of the residues Y113^QrcC^ and S138^QrcD^ of the QBS, and D70^QrcD^, Y110^QrcD^, and Y150^QrcD^ of the H^+^ pathway (Figure 4f), in the QrcABCD mechanism of energy conservation by deriving electrons and protons from opposite sides of the membrane to catalyze MK reduction. Different conserved residues appear to be involved in these functions in different enzymes containing the NrfC/PsrB and NrfD/PsrC proteins, which is in line with the diversity of reactions catalyzed by this family having very different thermodynamic profiles that can involve electroneutral, electrogenic or energy driven processes (Duarte et al. 2021). Further structural and physiological studies with different proteins will be important to understand the diversity of energy conservation mechanisms in the NrfD/PsrC/QrcD family.

## 4 METHODS

### 4.1 Construction of a homologous expression system for QrcABCD

Construction of the *N. vulgaris* Hildenborough *∆qrcABCD* strain was performed by homologous recombination, by transformation with a suicide plasmid—pMOGM1*‐∆qrcABCD*::KmR. This suicide plasmid was assembled through Sequence‐Ligation‐Independent Cloning (SLIC) (Li and Elledge 2012), joining four DNA fragments: the first with the upstream and initial sequence of *qrcA* (DVU0695*), the second with the kanamycin resistance cassette, the third with the final sequence (last 658 bp) of *qrcD* (DVU0692), and the last one a pUC backbone. The fragments were amplified through different PCR reactions using a high‐fidelity DNA polymerase. Specific primers containing homologous overhangs between fragments (Table S3) were employed to achieve hybridization. After plasmid propagation in *E. coli*, the pDNA was extracted and sequenced, and the suicide plasmid was transformed into *N. vulgaris* by electroporation (Keller et al. 2011). Recombinants were selected in anaerobic lactate/sulfate solid medium supplemented with 400 μg/mL of geneticin (G418). To confirm homologous recombination and interruption of the *qrcABCD* operon, genomic DNA was isolated through Wizard® Genomic DNA Purification Kit (Promega), followed by a PCR with primers *Fwd_qrcA* and Rev*_qrcD*_*stop* (Table S4) to confirm the size of the new modified operon for comparison with the native *qrc* operon.

For production of recombinant QrcABCD, an expression plasmid—pMOGM2‐*qrcABCD*‐strep‐tag containing the complete *qrcABCD* operon and a spectinomycin resistance cassette for selection was assembled using the SLIC protocol. For the plasmid assembly, three fragments were amplified, two that compose the backbone of the plasmid and another containing the *qrc* operon, using specific primers (Table S4). The ligation of these fragments was confirmed by hydrolysis of the assembled plasmid with *Xho*I, showing a linear fragment with the correct size, and the plasmid was sequenced for further confirmation. A Strep‐tag II sequence (TGGAGCCACCCCCAGTTCGAAAAG) was introduced in this plasmid in the 3′ side of the *qrcD* gene (upstream the STOP‐codon) by site‐directed mutagenesis (NZYMutagenisis Kit, NZYTech) using specific primers (Fwd_QrcDterm_add_Strep and Rev_QrcDterm_add_Strep) (Table S5). The expression plasmid, pMOGM2‐*qrcABCD*‐strep‐tag, was inserted into *∆qrcABCD* by electroporation as described earlier. Cells were left to recover at 37°C for 48 h in anaerobic lactate/sulfate‐rich medium (MOYLS4) and then plated on MOYLS4 solid‐medium with 400 μg/mL of G418 and 100 μg/mL of spectinomycin (Sm). The cultures were grown for 5 days, and colonies were inoculated in MOYLS4/G418/Sm selective media. For confirmation of the Qrc expression strain, plasmid DNA purification (NZYMiniprep Kit, NZYTech) was performed from each of these cultures and sequenced.

To construct the variants of QrcABCD, the expression plasmid (pMOGM2‐*qrcABCD*‐strep‐tag) was modified through site‐directed mutagenesis (NZYMutagenisis Kit, NZYTech), using specific primers designed for each mutation (Table S6). The modified plasmids were sequenced to confirm the mutations before electroporation into the *∆qrcABCD* strain.

### 4.2 Growth studies with genetically modified *N. vulgaris* strains

The modified *N. vulgaris* strains were grown anaerobically in modified MO basal medium (minimal media) (Zane et al. 2010), with different electron donors and acceptors to study the differences in their phenotypes. The pH of each medium was adjusted to 7.2 after the addition of all components as described in Zane et al. 2010 (Zane et al. 2010). Sodium sulfate (30 mM) was used as the terminal electron acceptor, while four electron donors were tested: lactate (60 mM), pyruvate (60 mM), formate (40 mM), and molecular hydrogen (H_2_, 2 bar). Bacterial growth was monitored by following the increase in optical density at 600 nm. For the pre‐inoculum, the cells were grown overnight in rich media (MOY, MO medium with 1 g/L of yeast extract) with lactate and sulfate as electron donor and terminal electron acceptor, respectively. After the first growth, cells were transferred to the defined media where they grew overnight. From these cultures, dilutions were made to obtain the same optical density and ensure that an equal number of cells was used as inoculum in different experiments. Thauers vitamins (1 mL/L of media) and spectinomycin were added to the medium before the final inoculum. Bacterial growth was performed in a sealed 96‐well plate, with constant agitation (237 cpm) in a final volume of 200 μL for at least 50 h, in a plate reader (Epoch 2, Biotek) installed inside an anaerobic chamber with a 98% N_2_ 2% H_2_ atmosphere. Bacterial growth with H_2_ as the energy source was performed in a final volume of 10 mL of liquid media in a 25 mL flask where the headspace was replaced by pure hydrogen and pressurized (2 bar) after inoculation and incubation at 37°C. Bacterial growth was monitored by the increase of the optical density at 600 nm. Each experimental point in growth curves is the average of the corresponding three independent biological replicates.

### 4.3 Production and purification of recombinant QrcABCD and variants

Cells were grown in modified Postgate medium C (Postgate 1984) containing 3.7 mM KH_2_PO_4_, 18.7 mM NH_4_Cl, 17.6 mM Na_2_SO_4_, 0.4 mM CaCl_2_·2H_2_O, 0.24 mM MgSO_4_·7H_2_O, 1 g/L yeast extract, 26 μM FeSO_4_·7H_2_O, 1 mM sodium citrate tribasic dihydrate, 0.57 mM L‐ascorbic acid, 0.88 mM sodium thioglycolate, 40 mM sodium formate, 10 mM sodium DL‐lactate and 100 mM Tris base, supplemented with 10 μM NiCl_2_·6H_2_O, 10 μM Na_2_SeO_3_·5H_2_O, 10 μM Na_2_O_4_W·2H_2_O, and 0.1 μM Na_2_MoO_4_·2H_2_O. Cells were collected by centrifugation, resuspended in 20 mM HEPES pH 7 buffer, containing DNase and protease inhibitors (cOmplete Mini EDTA‐free Protease Inhibitor, Roche), and disrupted in a French pressure cell in aerobic conditions. The crude extract was centrifuged at 8000*g* for 12 min at 4°C and, to isolate the membrane fraction, the supernatant was centrifuged at 109,000*g* for 2 h at 4°C. Membrane proteins were extracted by solubilization with 1.5% (w/v) n‐dodecyl‐β‐D‐maltoside (DDM) for 16 h at 4°C. The solubilized membrane proteins were loaded into a Strep‐tactin affinity column (Schmidt and Skerra 2007) previously equilibrated with 20 mM HEPES pH 7, 150 mM NaCl, 0.05% (w/v) DDM (Buffer A). The column was washed with five column volumes (cv) of Buffer A, and pure rQrcABCD was eluted with three cv of 20 mM HEPES pH 7, 150 mM NaCl, 0.05% (w/v) DDM, 50 mM D‐(+)‐biotin. Excess of D‐(+)‐biotin was removed by exchanging with buffer A. Purity of the eluted samples was assessed by 10% SDS‐Tricine PAGE stained with Coomassie blue for total protein and heme detection through heme‐staining. Recombinant QrcABCD proteins were also analyzed by Western blot in a 10% SDS‐Tricine PAGE with Strep‐tag II antibodies, and a 5–15% clear native (CN)‐PAGE, also stained with Coomassie and heme‐staining (Francis and Becker 1984). rQrcABCD concentration was determined by UV–vis spectrophotometry by the α‐peak (at 552 nm) upon reduction with sodium dithionite, using the previously determined ε_552nm_ = 113.6 mM^−1^ cm^−1^ (Venceslau et al. 2010).

### 4.4 Determination of aggregation temperature

The structural impact of the selected mutations was evaluated by analyzing the thermal stability of rQrcABCD variants in a Nanotemper Prometheus Panta. The proteins, at 1.5 mg mL^−1^ in Buffer A, were transferred to glass capillaries (10 μL per capillary) and placed in the sample holder. A linear temperature gradient (1°C min^−1^) between 25 and 95°C was applied and the thermal denaturation was monitored by the intrinsic tryptophan fluorescence (ITF‐DSF; excitation at 275 nm; emission at 330 and 350 nm), and thermal aggregation by light back‐reflection (turbidity) and dynamic light scattering (DLS). Data were collected in triplicates and are represented as average values ± standard deviation.

### 4.5 rQrcABCD reconstitution in liposomes

Pure fractions of recombinant QrcABCD WT and variants (Y113A^QrcC^, Y113F^QrcC^, D120A^QrcD^, S138A^QrcD^, D70A^QrcD^, D70H^QrcD^, Y150A^QrcD^, Y110A^QrcD^, R358A^QrcD^, and E413A^QrcD^) were reconstituted into MK4‐containing liposomes as previously reported (Venceslau et al. 2010). Briefly, MK4 was dissolved with L‐α‐phosphatidylcholine in chloroform and dried under N_2_ flux. The dried mixture was dissolved in 1 mM HEPES pH 7.5, 150 mM K_2_SO_4_, 200 μM Phenol Red and liposomes were prepared by extrusion with a 0.1 μm pore membrane. For each experiment, pure recombinant QrcABCD at a final concentration of 2.5 μM was mixed with the MK4‐containing liposomes, in the presence of 100 nM valinomycin and 0.006% (v/v) Triton X‐100. Incorporation of rQrcABCD into the liposomes occurs by removal of the detergent by incubation of the sample with 250 mg Bio‐Beads SM‐2 adsorbent (0.5 g/mL solution) for 1 h. The concentration of rQrcABCD in the proteoliposomes was measured through the full reduction of the complex with sodium dithionite. Total concentration of MK4 present within the proteoliposomes was determined spectroscopically, using ε_(270nm–290nm)_ = 14.6 mM^−1^ cm^−1^, after dissolving the liposomes in ethanol, solubilizing the MK4.

### 4.6 Stopped‐flow kinetic experiments with recombinant QrcABCD proteoliposomes

In anaerobic conditions, the different sets of proteoliposomes containing WT rQrcABCD or variants were diluted in 1 mM HEPES pH 7.5, 150 mM K_2_SO_4_ (previously degassed under argon) to achieve a final MK4 concentration of 20 μM in the presence of an O_2_ scavenging system—100 mM glucose, 0.75 nM catalase, 3.75 nM glucose oxidase, having a final concentration of rQrcABCD of 10–12 nM. The electron donor TpI*c*_3_ was purified as previously described (Duarte et al. 2018), diluted to 10 μM in the same buffer with the scavenging system and reduced to about 95% by following the α‐peak at 552 nm. Stopped flow experiments were performed at 30°C and electron transfer between reduced TpI*c*_3_ and MK4‐containing proteoliposomes was monitored by following absorbance at 552 nm, ε_(552nm)_ = 116 mM^−1^ cm^−1^ (Venceslau et al. 2010). At the same time, the protonation state of the phenol red was followed at 560 nm, an isosbestic point of TpI*c*_3_ (Duarte et al. 2018). All experiments were performed in steady‐state conditions relative to the Qrc complex and equivalent concentrations of MK4 and liposomes across all samples, with data from two biological and five technical replicates. To determine the unspecific direct reduction of MK4 by TpI*c*_3_, experiments with liposomes containing only MK4 were performed. rQrcABCD variants were considered to have a significant cytochrome *c*_3_:menaquinone oxidoreductase activity when the total oxidation rate of TpI*c*_3_ was considerably higher than the rate of its unspecific oxidation in the absence of the Qrc complex. Experiments were performed with at least two separate batches of proteoliposomes.

## AUTHOR CONTRIBUTIONS

**Gonçalo Manteigas:** Investigation; writing – review and editing; writing – original draft; validation. **Teresa Catarino:** Investigation; data curation; writing – review and editing. **João B. Vicente:** Investigation; data curation; writing – review and editing. **Américo G. Duarte:** Data curation; writing – original draft; writing – review and editing; validation; conceptualization; supervision; investigation; funding acquisition. **Inês A. C. Pereira:** Conceptualization; funding acquisition; writing – original draft; writing – review and editing; supervision; investigation.

## Supporting information


**Table S1.** Bacterial growth parameters of *N. vulgaris* Hildenborough WT, *∆qrcABCD* and complemented rQrc strains in different growth conditions. Growths were performed in defined media in a plate reader with sulfate and formate, pyruvate or lactate as electron donors and in Hungate tubes for H_2_/sulfate. μ_s_, specific growth rate; D_t_, cell doubling time; Max. OD_600_, maximum cell density measured at 600 nm. n.d., not determined.
**Table S2.** Growth parameters for *N. vulgaris* Hildenborough *∆qrcABCD*, rQrc and variants. Growth in defined media with formate/sulfate and lactate/sulfate monitored in a plate reader. μ_s_, specific growth rate; D_t_, cell doubling time; Max. OD_600_, maximum cell density measured at 600 nm. n.d., not determined.
**Table S3.** Primer sequences used for fragment amplification to construct pMOGM1‐*∆qrcABCD*::KmR plasmid. For each primer sequence the overhang and annealing region are highlighted in bold and underlined, respectively.
**Table S4.** Primer sequences used for fragment amplification to construct pMOGM2‐*qrcABCD* plasmid. For each primer sequence, the overhang regions and annealing regions are highlighted in bold and underlined, respectively.
**Table S5.** Primer sequences used for site‐directed mutagenesis of the pMOGM2‐*qrcABCD* plasmid to insert a Strep‐tag in the terminus of *qrcD*. For each primer sequence, the Strep‐affinity tag sequence is presented underlined.
**Table S6.** Primer sequences used for site‐directed mutagenesis of the pMOGM2‐*qrcABCD*‐strep plasmid to produce QrcABCD variants. For each primer sequence, the mutated sequence is presented in lowercase and underlined.
**Figure S1.** Construction of the *N. vulgaris ΔqrcABCD* deletion and complemented strains. (a) Agarose (1%) gel electrophoresis comparing the amplified DNA fragments of the native WT (band 1) and Δ*qrcABCD* strains (band 2), Marker‐Thermo Scientific™ GeneRuler 1 kb DNA ladder (left side). (b) Schematic representation of *N. vulgaris qrc* locus before and after gene modification with corresponding size in base pairs. (c) Schematic representation of the complementation vector used for the expression of rQrcABCD.
**Figure S2.** Bacterial growth curves of *N. vulgaris* strains. Comparison between the WT (pink triangles), *ΔqrcABCD* (black dots) and rQrc (blue circles) strains grown in lactate/sulfate (a), formate/sulfate (b), H_2_/sulfate (c) and pyruvate/sulfate (d). Each point is the average of three independent biological replicates.
**Figure S3.** Gel electrophoresis of rQrcABCD. (a) rQrcABCD analyzed in a 10% SDS‐PAGE with Coomassie staining, followed by heme‐staining, and Western blot detection of QrcD with anti‐Strep‐Tag antibodies. Marker‐NZYColour Protein Marker II (NZYTech). (b) rQrcABCD analyzed in a 5–15% Clear Native PAGE stained with Coomassie followed by heme staining, and Western blot detection of QrcD with anti‐Strep‐Tag antibodies. Marker‐High Molecular Weight (Cytiva).
**Figure S4.** Denaturing gel electrophoresis of rQrcABCD variants. Variants of rQrcABCD analyzed in a 10% SDS‐PAGE with Coomassie staining, followed by heme‐staining. Marker‐High Molecular Weight (Cytiva).
**Figure S5.** UV–Visible spectra of native and rQrcABCD. Spectra of native QrcABCD (a) and rQrcABCD (b), as purified (black) and reduced with excess of sodium dithionite (gray).
**Figure S6.** Thermal stability analysis of the rQrcABCD variants. The thermal stability of the expressed QrcABCD variants, determined through the midpoint of thermal aggregation analyzed by turbidometry, is shown as the difference in the average temperature relative to rQrcABCD. Data collected in triplicates and represented as average values ± standard deviation.
**Figure S7.** Internal pH changes for rQrcABCD variants that retain activity. Absorbance changes at 560 nm, after mixing reduced TpI*c*_3_ with proteoliposomes prepared with phenol red in the inner solution.
**Figure S8.** Phenol red calibration curve. Calibration curve of phenol red with tryptic hydrolysis of N‐α‐tosyl‐L‐arginyl‐O‐methylester (TAME), to convert phenol red absorbance at 560 nm to proton concentration. TAME solutions of different concentrations were mixed with a 200 μM trypsin solution prepared in 1 mM HEPES pH 7.5, 150 mM K_2_SO_4_, 200 μM phenol red (pH adjusted just before use).
**Figure S9.** Superposition of the structural model of QrcCD with different NrfCD homologs. Structural alignment of the QrcCD obtained using MODELLER (light blue) with an Alphafold 2 model of (a) PsrC from *W. succinogenes* (light pink), (b) HybB from *E. coli* (light orange), (c) DsmC from *E. coli* (green) (d) with the crystal structure of PsrBC from *T. thermophilus* (PDB: 2VPZ) (gray), and (e) with the cryoEM structure of ActC from *R. marinus* (PDB: 6F0K) (light pink). In all figures, a cartoon representation is used to depict the backbone structure of the protein evidencing relevant amino acids side chains represented by sticks with carbons colored in cyan for QrcD, in orange for PsrC from *W. succinogenes* (a) and HybB from *E. coli* (b), in green for DsmC from *E. coli*, in magenta for PsrB from *T. thermophilus* and ActC from *R. marinus*. Alphafold predictions were generated using the AlphaFold 3 Server (Google DeepMind). The models were produced using default parameters, with multiple sequence alignments automatically generated by the server internal pipeline using the UniRef100, MGnify, and BFD/MGnify databases.

## Data Availability

The data that support the findings of this study are available on request from the corresponding author. The data are not publicly available due to privacy or ethical restrictions.
