# Supplementary material for: Linking NrfD/PsrC‐like architecture to energy conservation: Functional residues in the quinone reactive QrcABCD complex of sulfate‐reducing bacteria
Source: Protein Sci. 2026 Apr 8;35(5):e70557. doi: 10.1002/pro.70557 (PMC13059093; doi:10.1002/pro.70557)
Supplement: Supplementary file 1 — Table S1. Bacterial growth parameters of N. vulgaris Hildenborough WT, ∆qrcABCD and complemented rQrc strains in different growth conditions. Growths were performed in defined media in a plate reader with sulfate and formate, pyruvate or lactate as electron donors and in Hungate tubes for H2/sulfate. μs, specific growth rate; Dt, cell doubling time; Max. OD600, maximum cell density measured at 600 nm. n.d., not determined. Table S2. Growth parameters for N. vulgaris Hildenborough ∆qrcABCD, rQrc and variants. Growth in defined media with formate/sulfate and lactate/sulfate monitored in a plate reader. μs, specific growth rate; Dt, cell doubling time; Max. OD600, maximum cell density measured at 600 nm. n.d., not determined. Table S3. Primer sequences used for fragment amplification to construct pMOGM1‐∆qrcABCD::KmR plasmid. For each primer sequence the overhang and annealing region are highlighted in bold and underlined, respectively. Table S4. Primer sequences used for fragment amplification to construct pMOGM2‐qrcABCD plasmid. For each primer sequence, the overhang regions and annealing regions are highlighted in bold and underlined, respectively. Table S5. Primer sequences used for site‐directed mutagenesis of the pMOGM2‐qrcABCD plasmid to insert a Strep‐tag in the terminus of qrcD. For each primer sequence, the Strep‐affinity tag sequence is presented underlined. Table S6. Primer sequences used for site‐directed mutagenesis of the pMOGM2‐qrcABCD‐strep plasmid to produce QrcABCD variants. For each primer sequence, the mutated sequence is presented in lowercase and underlined. Figure S1. Construction of the N. vulgaris ΔqrcABCD deletion and complemented strains. (a) Agarose (1%) gel electrophoresis comparing the amplified DNA fragments of the native WT (band 1) and ΔqrcABCD strains (band 2), Marker‐Thermo Scientific™ GeneRuler 1 kb DNA ladder (left side). (b) Schematic representation of N. vulgaris qrc locus before and after gene modification with corresponding siz [file PRO-35-e70557-s001.docx]

**Linking NrfD/PsrC-like Architecture to Energy Conservation: Functional Residues in the quinone reactive QrcABCD Complex of sulfate reducing bacteria**

Gonçalo Manteigas^1^, Teresa Catarino^1,2^, João B. Vicente^1^, Américo G. Duarte^1^*, Inês A. C. Pereira^1^*

^1^Instituto de Tecnologia Química e Biológica António Xavier / Universidade Nova de Lisboa, Av. da República, Estação Agronómica Nacional, 2780-157 Oeiras, Portugal

^2^Departamento de Química, Faculdade de Ciências e Tecnologia, Universidade Nova de Lisboa, 2829-516, Caparica, Portugal

SUPPLEMENTARY INFORMATION

*Corresponding authors:

Inês A. C. Pereira

[ipereira@itqb.unl.pt](mailto:ipereira@itqb.unl.pt)

and

Américo G. Duarte

[americoduarte@itqb.unl.pt](mailto:americoduarte@itqb.unl.pt)

Phone: +351 21 4469327;

**Table S1 – Bacterial growth parameters of *N.* *vulgaris* Hildenborough WT, *∆qrcABCD* and complemented rQrc strains in different growth conditions.** Growths were performed in defined media in a plate reader with sulfate and formate, pyruvate or lactate as electron donors and in Hungate tubes for H_2_/sulfate. μ_s_– Specific growth rate; D_t_ – Cell doubling time; Max. OD_600_ – maximum cell density measured at 600 nm. n.d. – not determined.

|  | **Formate/sulfate** | | | **H_2_/sulfate** | | | **Pyruvate/sulfate** | | | **Lactate/sulfate** | | |
| --- | --- | --- | --- | --- | --- | --- | --- | --- | --- | --- | --- | --- |
|  | **μ_s_**  **(h^-1^)** | **D_t_ (h)** | **Max. OD_600nm_** | **μ_s_**  **(h^-1^)** | **D_t_ (h)** | **Max. OD_600nm_** | **μ_s_**  **(h^-1^)** | **D_t_ (h)** | **Max. OD_600nm_** | **μ_s_**  **(h^-1^)** | **D_t_ (h)** | **Max. OD_600nm_** |
| **WT** | 0.069 | 10.0 | 0.43 | 0.127 | 5.5 | 0.43 | 0.041 | 16.8 | 0.33 | 0.190 | 3.7 | 0.51 |
| ***ΔqrcABCD*** | n.d. | n.d. | 0.10 | n.d. | n.d. | 0.11 | n.d. | n.d. | 0.10 | 0.184 | 3.8 | 0.45 |
| **rQrc** | 0.064 | 10.8 | 0.34 | 0.099 | 7.0 | 0.42 | 0.043 | 16.3 | 0.34 | 0.196 | 3.6 | 0.48 |

**Table S2 – Growth parameters for *N.* *vulgaris* Hildenborough *∆qrcABCD*, rQrc and variants.** Growth in defined media with formate/sulfate and lactate/sulfate monitored in a plate reader. μ_s_– Specific growth rate; D_t_ – Cell Doubling Time; Max. OD_600_ – maximum cell density measured at 600 nm. n.d. – not determined.

|  | **Formate/Sulfate** | | | **Lactate/Sulfate** | | |
| --- | --- | --- | --- | --- | --- | --- |
|  | **μ_s_**  **(h^-1^)** | **D_t_ (h)** | **Max. OD_600nm_** | **μ_s_**  **(h^-1^)** | **D_t_ (h)** | **Max. OD_600nm_** |
| **rQrc** | 0.071 | 9.8 | 0.36 | 0.235 | 3.0 | 0.54 |
| ***ΔqrcABCD*** | n.d | n.d | 0.09 | 0.221 | 3.1 | 0.53 |
| **Y113A^QrcC^** | n.d | n.d | 0.10 | 0.188 | 3.7 | 0.43 |
| **Y113F^QrcC^** | n.d | n.d | 0.08 | 0.225 | 3.1 | 0.55 |
| **S138A^QrcD^** | n.d | n.d | 0.08 | 0.161 | 4.3 | 0.56 |
| **D70A^QrcD^** | n.d | n.d | 0.08 | 0.235 | 2.9 | 0.47 |
| **D70H^QrcD^** | n.d | n.d | 0.08 | 0.237 | 2.9 | 0.51 |
| **Y150A^QrcD^** | n.d | n.d | 0.07 | 0.232 | 3.0 | 0.51 |
| **Y110A^QrcD^** | n.d | n.d | 0.08 | 0.244 | 2.8 | 0.47 |
| **R358A^QrcD^** | 0.061 | 11.4 | 0.31 | 0.255 | 2.7 | 0.48 |
| **E413A^QrcD^** | 0.062 | 11.1 | 0.25 | 0.236 | 2.9 | 0.49 |
| **D120A^QrcD^** | 0.069 | 10.0 | 0.22 | 0.259 | 2.7 | 0.56 |

**Table S3 - Primer sequences used for fragment amplification to construct pMOGM1-*∆qrcABCD*::KmR plasmid.** For each primer sequence the overhang and annealing region are highlighted in bold and underlined, respectively.

| Primer | Sequence (5’ → 3’) |
| --- | --- |
| IP11:Fwd_SmR | **CCTTCGATCAGATGTTCACC**CCAGCCAGGACAGAAATGCCTCG |
| IP12:Rev_pUC_ori | ATGTGAGCAAAAGGCCAGCAAAAGGC |
| Fwd_QrcA*_*upstream | **TTTTGCTCACAT**CCTGCGTTTGACATGACGC |
| Rev_QrcA | **CGTCATGTAAGCCCA**GAACTGGTGACACTGGTTGCAG |
| Fwd_Prom Kan | **CAGTGTCACCAGTTC**TGGGCTTACATGACGATAGC |
| Rev_Prom Kan | **ATGTTCAGGATGTAG**TGCTGGTTTCCTGGATGCC |
| Fwd_QrcD | **TCCAGGAAACCAGCA**CTACATCCTGAACATCGACC |
| Rev_QrcD | **TTCTGTCCTGGCTGG**GGTGAACATCTGATCGAAGG |

**Table S4 - Primer sequences used for fragment amplification to construct pMOGM2-*qrcABCD* plasmid.**  For each primer sequence, the overhang regions and annealing regions are highlighted in bold and underlined, respectively.

| Primer | Sequence (5’ → 3’) |
| --- | --- |
| Fwd_pMO_ori | **GGGAACTGCCAGGCATCAA**ATAAAACGAAAGGCTCAGTCG |
| Rev_pMO_PromKan | **CTGCCTGTCCTCCAT**ATGGTACCTCCTGGGACTGC |
| Fwd_*qrcA* | **CCCAGGAGGTACCAT**ATGGAGGACAGGCAGTTAAC |
| Rev_*qrcD_stop* | **ATCGGGTCTTTTCGTT**CGCGCAAGGGTTACTTGC |
| Fwd_pBG1 | **GTAACCCTTGCGCG**AACGAAAAGACCCGATCATGA |
| Rev_pBG1 | **CGACTGAGCCTTTCGTTTTAT**TTGATGCCTGGCAGTTC |

**Table S5- Primer sequences used for site-directed mutagenesis of the pMOGM2-*qrcABCD* plasmid to insert a Strep-tag in the terminus of *qrcD.*** For each primer sequence, the Strep-affinity tag sequence is presented underlined.

| Primer | Sequence (5’ → 3’) |
| --- | --- |
| Fwd_QrcDterm_add_Strep | AGTTGAACCGCAAGTGGAGCCACCCCCAGTTCGAAAAGTAACCCTTGCGCGAGCATCGC |
| Rev_QrcDterm_add_Strep | CGATGCTCGCGCAAGGGTTACTTTTCGAACTGGGGGTGGCTCCACTTGCGGTTCAACTCCG |

**Table S6 - Primer sequences used for site-directed mutagenesis of the pMOGM2-*qrcABCD*-strep plasmid to produce QrcABCD variants*.*** For each primer sequence, the mutated sequence is presented in lowercase and underlined.

| Mutation | Primer | Sequence (5’ → 3’) |
| --- | --- | --- |
| Y113A^QrcC^ | Fwd_mut_QrcC(Y113A) | GATGCATCGGTTGCCGGgccTGCATGGCCTCGTGCC |
|  | Rev_mut_QrcC(Y113A) | GGCACGAGGCCATGCAggcCCGGCAACCGATGCATC |
| Y113F^QrcC^ | Fwd_mut_QrcC(Y113F) | GATGCATCGGTTGCCGGttcTGCATGGCCTCGTGCC |
|  | Rev_mut_QrcC(Y113F) | GGCACGAGGCCATGCAgaaCCGGCAACCGATGCATC |
| D120A^QrcD^ | Fwd_mut_QrcD(D120A) | CATGCTCGTGCTCGTGCTCgccATCGGCCAGCCGCTTC |
|  | Rev_mut_QrcD(D120A) | GAAGCGGCTGGCCGATggcGAGCACGAGCACGAGCATG |
| S138A^QrcD^ | Fwd_mut_QrcD(S138A) | GCACGCCAACGTGCACgccATGCTCACCGAAGTCATC |
|  | Rev_mut_QrcD(S138A) | GATGACTTCGGTGAGCATggcGTGCACGTTGGCGTGC |
| D70A^QrcD^ | Fwd_mut_QrcD(D70A) | CCTCTGGATCACGTTCgccCTTGCCGTCATCGCCC |
|  | Rev_mut_QrcD(D70A) | GGGCGATGACGGCAAGggcGAACGTGATCCAGAGG |
| D70H^QrcD^ | Fwd_mut_QrcD(D70H) | CCTCTGGATCACGTTCcacCTTGCCGTCATCGCCC |
|  | Rev_mut_QrcD(D70H) | GGGCGATGACGGCAAGgtgGAACGTGATCCAGAGG |
| Y150A^QrcD^ | Fwd_mut_QrcD(Y150A) | CATCTTCTGCATCACGTGCgccTGCCTCGTGCTCATCATC |
|  | Rev_mut_QrcD(Y150A) | GATGATGAGCACGAGGCAggcGCACGTGATGCAGAAGATG |
| Y110A^QrcD^ | Fwd_mut_QrcD(Y110A) | CATCGGCTTTCTGTGCgccTCGGGTGCCATGCTCG |
|  | Rev_mut_QrcD(Y110A) | CGAGCATGGCACCCGAggcGCACAGAAAGCCGATG |
| R358A^QrcD^ | Fwd_mut_QrcD(R358A) | CGGCATCACCATCAACgccTACGTCATGACCGTGC |
|  | Rev_mut_QrcD(R358A) | GCACGGTCATGACGTAggcGTTGATGGTGATGCCG |
| E413A^QrcD^ | Fwd_mut_QrcD(E413A) | CTTCCCGCAggcAGCGGAGTTGAACC |
|  | Rev_mut_QrcD(E413A) | GGTTCAACTCCGCTgccTGCGGGAAG |

**Figure S1** – **Construction of the *N. vulgaris* *ΔqrcABCD* deletion and complemented strains.** (A) Agarose (1 %) gel electrophoresis comparing the amplified DNA fragments of the native WT (band 1) and Δ*qrcABCD* strains (band 2), Marker - Thermo Scientific™ GeneRuler 1 kb DNA ladder (left side). (B) Schematic representation of *N. vulgaris* *qrc* locus before and after gene modification with corresponding size in base pairs. (C) Schematic representation of the complementation vector used for the expression of rQrcABCD.


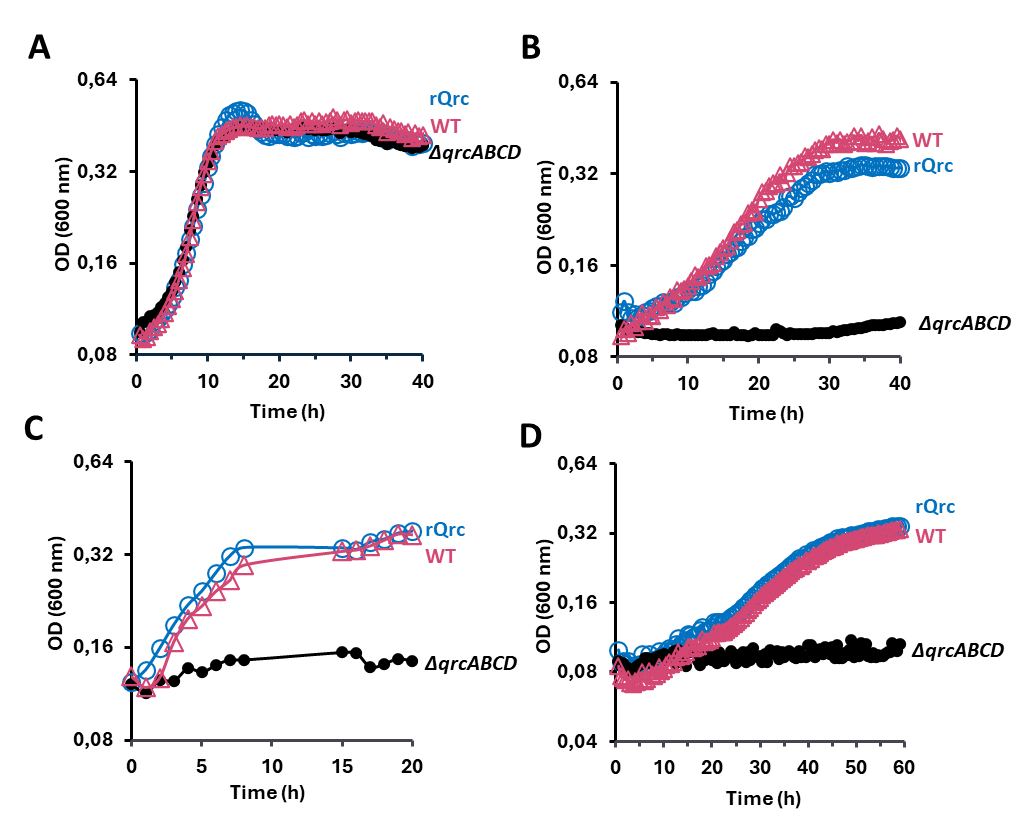


**Figure S2 – Bacterial growth curves of *N. vulgaris* strains.** Comparison between the WT (pink triangles), *ΔqrcABCD* (black dots) and rQrc (blue circles) strains grown in lactate/sulfate (A), formate/sulfate (B), H_2_/sulfate (C) and pyruvate/sulfate (D). Each point is the average of three independent biological replicates.


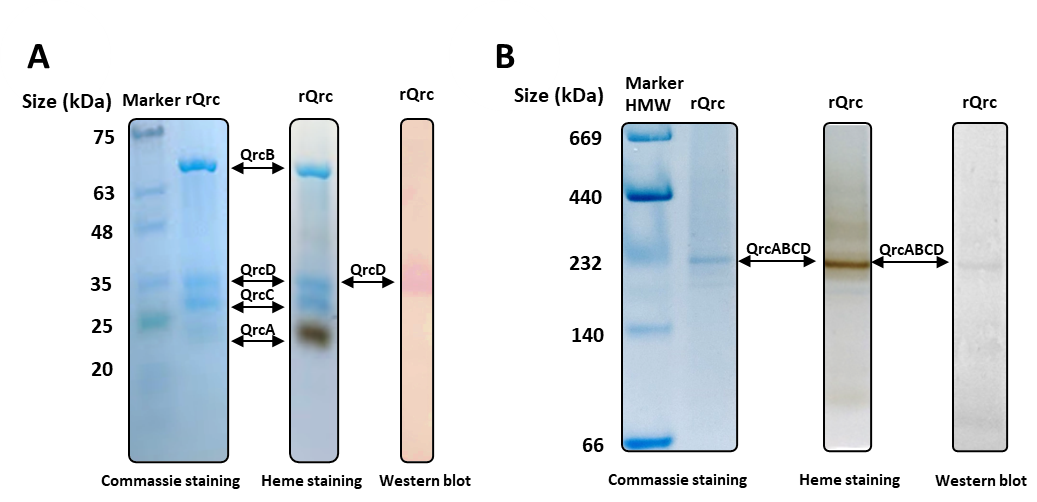


**Figure S3 – Gel electrophoresis of rQrcABCD.** (A) rQrcABCD analyzed in a 10 % SDS-PAGE with Coomassie staining, followed by heme-staining, and Western blot detection of QrcD with anti-Strep-Tag antibodies. Marker - NZYColour Protein Marker II (NZYTech). (B) rQrcABCD analyzed in a 5-15% Clear Native PAGE stained with Coomassie followed by heme staining, and Western blot detection of QrcD with anti-Strep-Tag antibodies. Marker - High Molecular Weight (Cytiva).


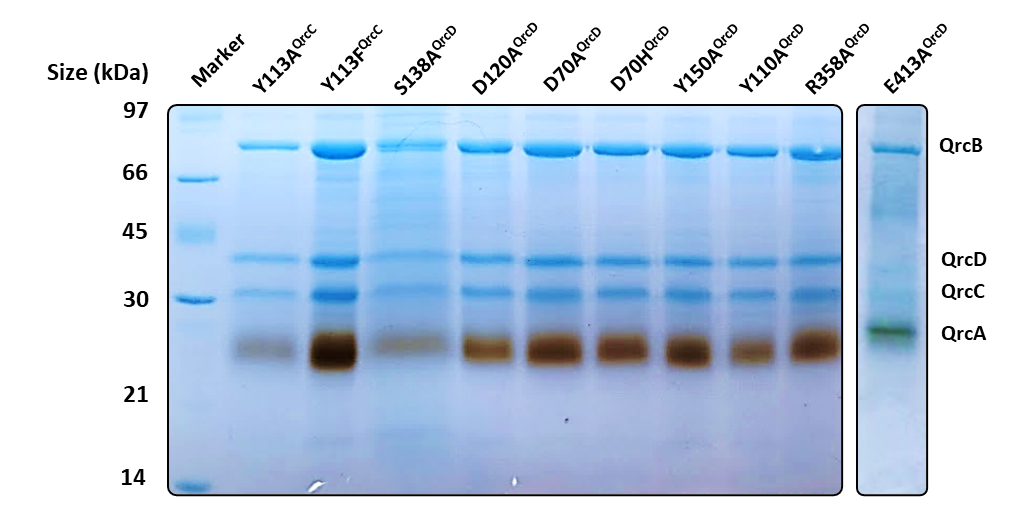


**Figure S4** – **Denaturing** **gel electrophoresis of rQrcABCD variants.** Variants of rQrcABCD analyzed in a 10% SDS-PAGE with Coomassie staining, followed by heme-staining. Marker - High Molecular Weight (Cytiva).


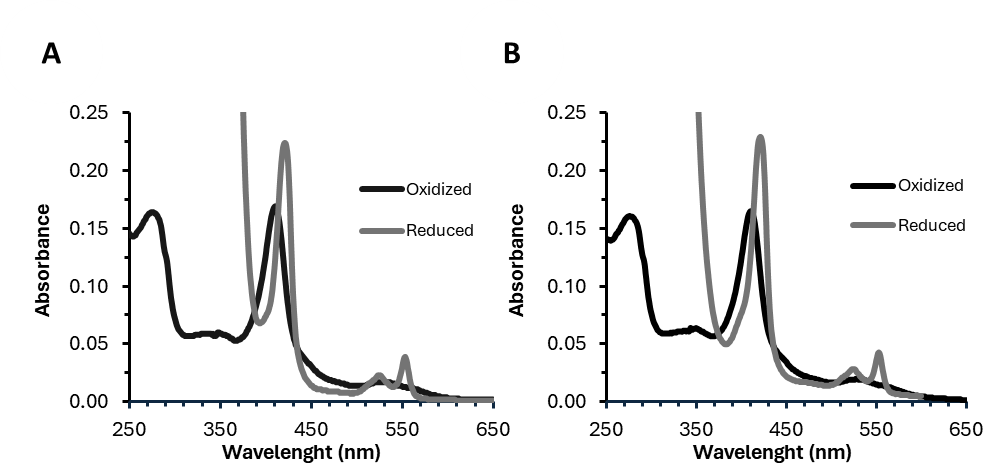


**Figure S5 – UV-Visible spectra of native and rQrcABCD.** Spectra of native QrcABCD (A) and rQrcABCD (B), as purified (black) and reduced with excess of sodium dithionite (gray).


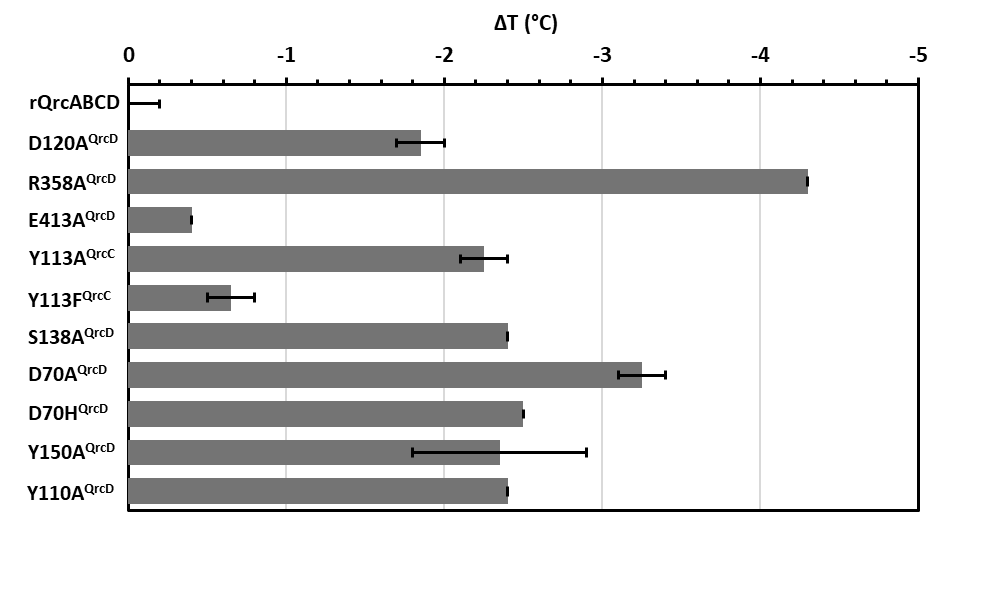


**Figure S6 - Thermal stability analysis of the rQrcABCD variants.** The thermal stability of the expressed QrcABCD variants, determined through the midpoint of thermal aggregation analyzed by turbidometry, is shown as the difference in the average temperature relative to rQrcABCD. Data collected in triplicates and represented as average values ± standard deviation.

…………………………….


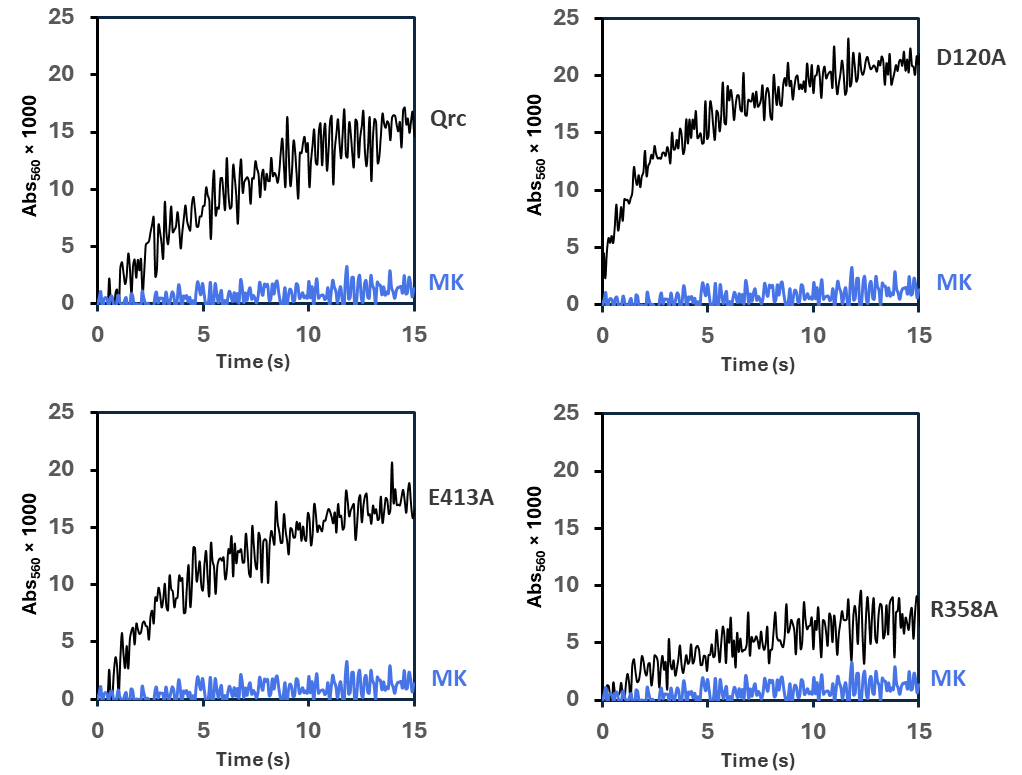


**Figure S7 – Internal pH changes for rQrcABCD variants that retain activity.** Absorbance changes at 560 nm, after mixing reduced TpI*c*_3_ with proteoliposomes prepared with phenol red in the inner solution.

**Figure S8 – Phenol red calibration curve**. Calibration curve of phenol red with tryptic hydrolysis of N-α-tosyl-L-arginyl-O-methylester (TAME), to convert phenol red absorbance at 560 nm to proton concentration. TAME solutions of different concentrations were mixed with a 200 μM trypsin solution prepared in 1 mM HEPES pH 7.5, 150 mM K_2_SO_4_, 200 μM phenol red (pH adjusted just before use).

**Figure S9 – Superposition of the structural model of QrcCD with different NrfCD homologs.** Structural alignment of the QrcCD obtained using MODELLER (light blue) with an Alphafold 2 model of (A) PsrC from *W. succinogenes* (light pink), (B) HybB from *E. coli* (light orange), (C) DsmC from *E. coli* (green) (D) with the crystal structure of PsrBC from *T. thermophilus* (PDB: 2VPZ) (grey), and (E) with the cryoEM structure of ActC from *R. marinus* (PDB: 6F0K) (light pink). In all figures, a cartoon representation is used to depict the backbone structure of the protein evidencing relevant amino acids side chains represented by sticks with carbons colored in cyan for QrcD, in orange for PsrC from *W. succinogenes* (A) and HybB from *E. coli* (B), in green for DsmC from *E. coli*, in magenta for PsrB from *T. thermophilus* and ActC from *R. marinus.*  Alphafold predictions were generated using the AlphaFold 3 Server (Google DeepMind). The models were produced using default parameters, with multiple sequence alignments automatically generated by the server internal pipeline using the UniRef100, MGnify, and BFD/MGnify databases.
